# Analysis of influencing factors on soil Zn content using generalized additive model

**DOI:** 10.1038/s41598-018-33745-9

**Published:** 2018-10-22

**Authors:** Yan Jiang, Wen-Wu Gao, Jin-Ling Zhao, Qian Chen, Dong Liang, Chao Xu, Lin-Sheng Huang, Li-Min Ruan

**Affiliations:** 1 School of Economics, Anhui University, Hefei, 230601 China; 2 National United Engineering Research Center for Analysis and Application of Agro-Ecological Big Data, Anhui University, Hefei, 230601 China

## Abstract

Soil zinc (Zn) plays a crucial role in plant growth, but excessive accumulation in the environment may lead to air, water and soil pollution. It is affected by various chemical, environmental and spatial factors. Therefore, it is important to identify the factors influencing Zn content in the landscape. The main motivation for this study is to determine the suitability of a generalized additive model (GAM) to describe change in soil Zn content due to influencing factors. A total of 1497 soil nutrient samples were collected in Fangshan District, Beijing, China. Organic matter (OM), available phosphorus (AP), available potassium (AK), alkali-hydrolyzed nitrogen (AHN) and slowly available potassium (SAK) are considered. The relationship between Zn, nutrients and geographic location (latitude & longitude) is investigated using the GAM. More precisely, the Akaike information criterion (AIC) is used to select influencing factors on Zn content and cross-validated to avoid overfitting of the multivariate model. The results show that Zn content reaches its maximum at latitude 39.8°N and longitude 115.9°E. Zinc content increases as AP increases to 150 mg/kg. When OM content is greater than 90 g/kg, Zinc content decreases with an increase in OM content. Factors that affected Zn content, in descending order of significance derived from deviance explained and adjustment coefficient of determination (Adj.*R*^2^) were AP, latitude, AHN, AK and OM. Moreover, the interactions between latitude and longitude, AHN and AP, OM and AK have significant impact on Zn.

## Introduction

Zinc (Zn) in soil is one of the essential trace elements of plants^1–3^. When a plant is short of Zn, the growth in the stem and bud is reduced, and normal growth will be significantly affected. Similarly, Zn is required in the photosynthesis cycle^4^. Nevertheless, various influencing factors can affect the accumulation of Zn. For example, geographic location (e.g., latitude & longitude) has influenced the distribution and content of vegetation, soil nutrients and heavy metals^5–11^. Moreover, a considerable number of interactions are taking place in the soil between physical and chemical properties, such as, organic matter (OM), soil reaction (pH), calcium carbonate (CaCO_3_) and essential macro and micronutrients (P, K, Ca, Mg, Mn, Fe, Zn, and Cu)^12,13^.

Previous studies investigating Zn in soils have mainly focused on the influence of Zn on plants^14^, Zn content prediction^15^, Zn pollution characteristics^16^, source analysis^17^ and potential ecological risk assessment^18^. Furthermore, spatial analysis and statistical methods, such as multivariate analysis^19–21^ have been used to analyze the relationships of nutrients and heavy metals on plant and soils. But the nonlinear relationship between soil nutrients and heavy metal elements in soils has not been identified. Moreover, the effect of interactions of influencing factors on variation of soil Zn content should be also figured out. The interactions have an important impact on the material circulation in the soil circle, and it is also important for maintaining the ecological balance of environmental materials and eliminating pollutants to soil, plants and even humans^22^. This aims to identify the interactions of geographical and physical factors that could affect Zn content in soil using a non-parametric model, such as generalized additive model (GAM).

GAM has been widely used in medical application^23–25^, financial research^26^, fishery survey^27^ and environmental and climate studies^28–30^, due to specific advantages^31,32^. For example, it can directly deal with the nonlinear relationship between response variables and multiple explanatory variables^33^, especially for analysis of large data sets^34^. Furthermore, GAM can be used to analyse interactions between influencing factors on the response variable^35–37^. Conversely, traditional statistical methods cannot perform well in addressing the complex nonlinear relationship^38,39^. In our study, soil Zn content (here after referred to as Zn) is taken as an example to study the relationship between soil heavy metals, nutrients and geographic location (latitude & longitude). In the GAM, Zn is used as the response variable, geographic location and five types of soil nutrients are used as the explanatory variables.

## Results

### Pre-analysis of selected variables

The normal distribution is rejected at the significant level of 5% depending on the Shapiro-Wilk test. It does not meet the data requirement for binomial distribution. Consequently, the log function is selected as the link function^33,37^. It is found that most explanatory variables could pass the significance test at the *P-*value < 0.01 level, but most of the Pearson correlation coefficients (*R*s) are less than 0.3 (Table 1). The correlations are low among the variables. The largest *R* is between SAK and AK with the value of 0.443, which shows that the correlation is high in the large number of samples.

**Table 1:** Comparison of the *R* among the explanatory variables.

	Latitude	Longitude	OM	AHN	AP	SAK	AK
Latitude	1	0.159**	0.233**	0.166**	−0.105**	−0.032	0.085**
Longitude	0.159**	1	−0.349**	−0.220**	−0.131**	−0.152**	−0.072*
OM	0.233**	−0.349**	1	0.354**	0.099**	0.140**	0.211**
AHN	0.166**	−0.220**	0.354**	1	0.181**	0.250**	0.199**
AP	−0.105**	−0.131**	0.099**	0.181**	1	0.225**	0.173**
SAK	−0.032	−0.152**	0.140**	0.250**	0.225**	1	0.443**
AK	0.085**	−0.072*	0.211**	0.199**	0.173**	0.443**	1

**Indicates significant differences at the probability level of 0.01; *Indicates significant differences at the probability level of 0.05.

### Univariate analysis of influences on Zn

The regression model with cubic splines is used to analyze the influences of each individual explanatory variable on Zn and corresponding fitting degree of the model (Table 2). The results show that all the seven explanatory variables have passed the significance test at the *P-*value < 0.01 level, suggesting that each individual variable is statistically significant for the influence of Zn, with a low deviance explained. The deviance explained of AP and longitude are higher with the values of 20.2% and 16.5%, respectively. The corresponding adjustment coefficient of determination (Adj.*R*^2^) which increases with the increase in the number of independent variables are 0.16 and 0.2 for AP and longitude, respectively. The precision of model derived from each individual explanatory variable is low. Consequently, multiple variable interactions are considered for investigating their influences on Zn.

**Table 2:** Test of the GAM using univariate analysis.

Smoothing effect	Edf	Ref.df	*F-*value	*P-*value	Deviance explained (%)	Adj.*R*^2^
S(latitude)	4.912	6.015	6.827	3.71e–07***	2.99	0.027
S(longitude)	8.318	8.872	31.58	<2e–16***	16.5	0.160
S(OM)	6.688	7.811	13.56	<2e–16***	7.03	0.066
S(AHN)	6.824	7.926	15.32	<2e–16***	7.94	0.075
S(AP)	5.812	6.905	53.03	<2e–16***	20.2	0.200
S(SAK)	3.452	4.368	13.15	4.78e–11***	3.97	0.037
S(AK)	8.038	8.753	13.93	<2e–16***	7.84	0.073

***Indicates *P-*value < 0.01 level.

### Multivariate analysis of influences on Zn

The variables are gradually added to the GAM, and the tests are carried out using the Akaike information criterion (AIC) score (Table 3). It can be found that the AIC scores are generally reduced with the gradual increase of variables. Conversely, when SAK is added, the score increases by about 0.6, and the P-value is 0.031. SAK does not pass the significance test at the P-value < 0.01 level, which indicates that SAK has little effect on Zn. The other variables of latitude, longitude, OM, AHN, AP and AK significantly affected the changes of Zn at the P-value < 0.01 level. When all factors are added, the Adj.*R*^2^ = 0.4

**Table 3:** Test of the GAM using multivariate analysis.

Index	S(latitude)	S(longitude)	S(OM)	S(AHN)	S(AP)	S(SAK)	S(AK)
EDF	7.342	8.807	2.620	7.082	3.022	1.000	5.155
Ref.df	8.332	8.987	3.389	8.151	3.759	1.000	6.293
AIC	5796.933	5521.656	5451.596	5414.611	5183.496	5184.049	5181.697
*F-*value	13.048	23.945	4.094	5.241	47.390	4.648	5.157
*P*-value	<2e–16***	<2e–16***	0.00469***	1.41e–06***	<2e–16***	0.0312	1.96e–05***

***Indicates *P-*value < 0.01 level.

### Model fitting after concurvity diagnosis analysis

**Figure 1:**
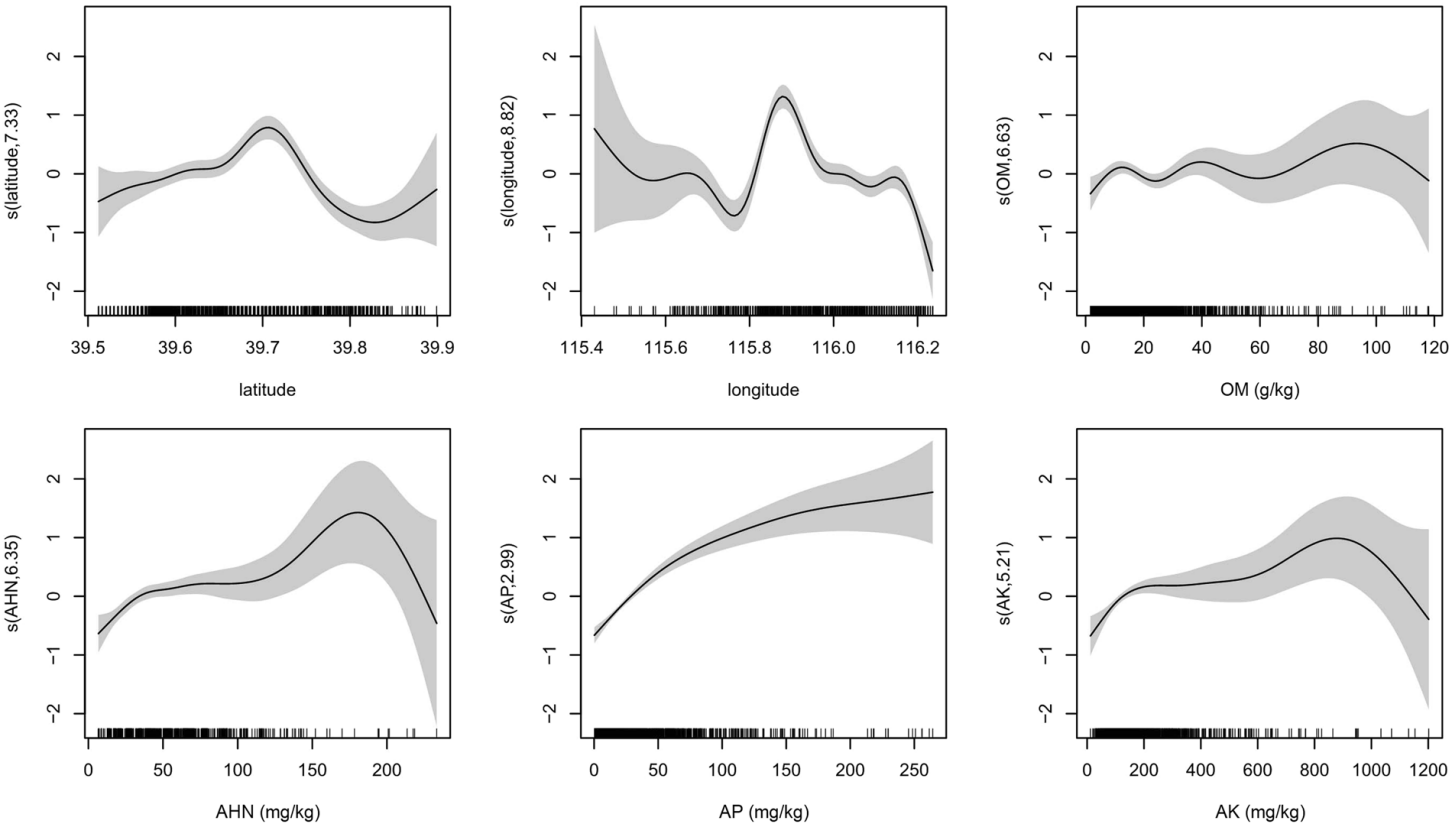
Estimated smoothness of six variables on Zn; *y*-axis is the partial effect of the variable and shadow section is the standard-error confidence intervals.

Three index values of S(SAK) and S(AK) are all close to or greater than 0.5 (Table 4), suggesting that they have a concurvity (a correlation between S(SAK) and S(AK)). Combining the results of concurvity of SAK and the multivariate analysis, SAK was removed from the model. After refitting the model in the absence of SAK, it identified that AHN, AK, AP, longitude, latitude significantly influenced Zn (Table 5). The refitted GAM with the deviance explained of 70.4% and Adj.*R*^2^ of 0.6 is an improvement on the model which does not have concurvity. The refitted GAM identifies the effect of the influencing factors on the changes in Zn content (Fig. 1) and the resulting nonlinear relationships (EDF ≠ 1). The model predicts that Zn content increases with the rise in latitude, peaking at 39.7°N. Zn reaches the maximum at longitude 115.9°E and 39.8°N, and it has little change with the change of OM content.

**Table 4:** Test of the concurvity of the smooth function.

Project	Parameter	S(latitude)	S(longitude)	S(OM)	S(AHN)	S(AP)	S(SAK)	S(AK)
Worst	6.62271e–22	0.570	0.549	0.405	0.471	0.286	0.639	0.854
Observed	6.62271e–22	0.200	0.189	0.372	0.319	0.262	0.584	0.695
Estimate	6.62271e–22	0.346	0.376	0.147	0.354	0.168	0.489	0.419

**Table 5:** Hypothesis test of the refitted GAM.

Index	S(latitude)	S(longitude)	S(OM)	S(AHN)	S(AP)	S(AK)
EDF	7.375	8.790	2.688	7.167	2.902	5.056
Ref.df	8.354	8.985	3.475	8.216	3.614	6.184
*F-*value	12.830	23.643	4.363	4.910	47.771	4.473
*P*-value	<2e–16***	<2e–16***	0.00285***	4.03e–06***	<2e–16***	0.0001***

***Indicates *P-*value < 0.01 level.

### Cross-validation of the refitted multivariate GAM

To avoid overfitting, cross-validation was used to test the refitted multivariate GAM. The difference between the predicted value and the measured value was small, and the six variables passed the significance test at the *P-*value < 0.01 level (Table 6). The optimal model can reasonably reflect the influencing factors on Zn.

**Table 6:** Cross-validation of GAM based Zn variation.

Index	Latitude	Longitude	OM	AHN	AP	AK
*F*-value	7.593	14.276	4.187	17.880	36.804	12.643
*P*-value	1.79e–09***	<2e–16***	0.001007**	2.57e–05***	<2e–16***	0.000395***

***Indicates *P-*value < 0.01 level.

### Interactions of multivariate factors on Zn

The model deviance explained derived from GAM was 72.1%, with the Adj.*R*^2^ of 0.63. The estimated degree of freedom of the longitude-HN interaction was 1 (Table 7). The *F*-value for the longitude-latitude interaction, the HN-AP interaction and the OM-AK interaction are 19.857, 4.678 and 4.433, respectively. These interactions passed the significance test at the *P*-value < 0.01 level. Similarly, the latitude-AP interaction, the latitude-OM interaction and the latitude-AK interaction passed the significance test at the *P*-value < 0.05 level.

**Figure 2:**
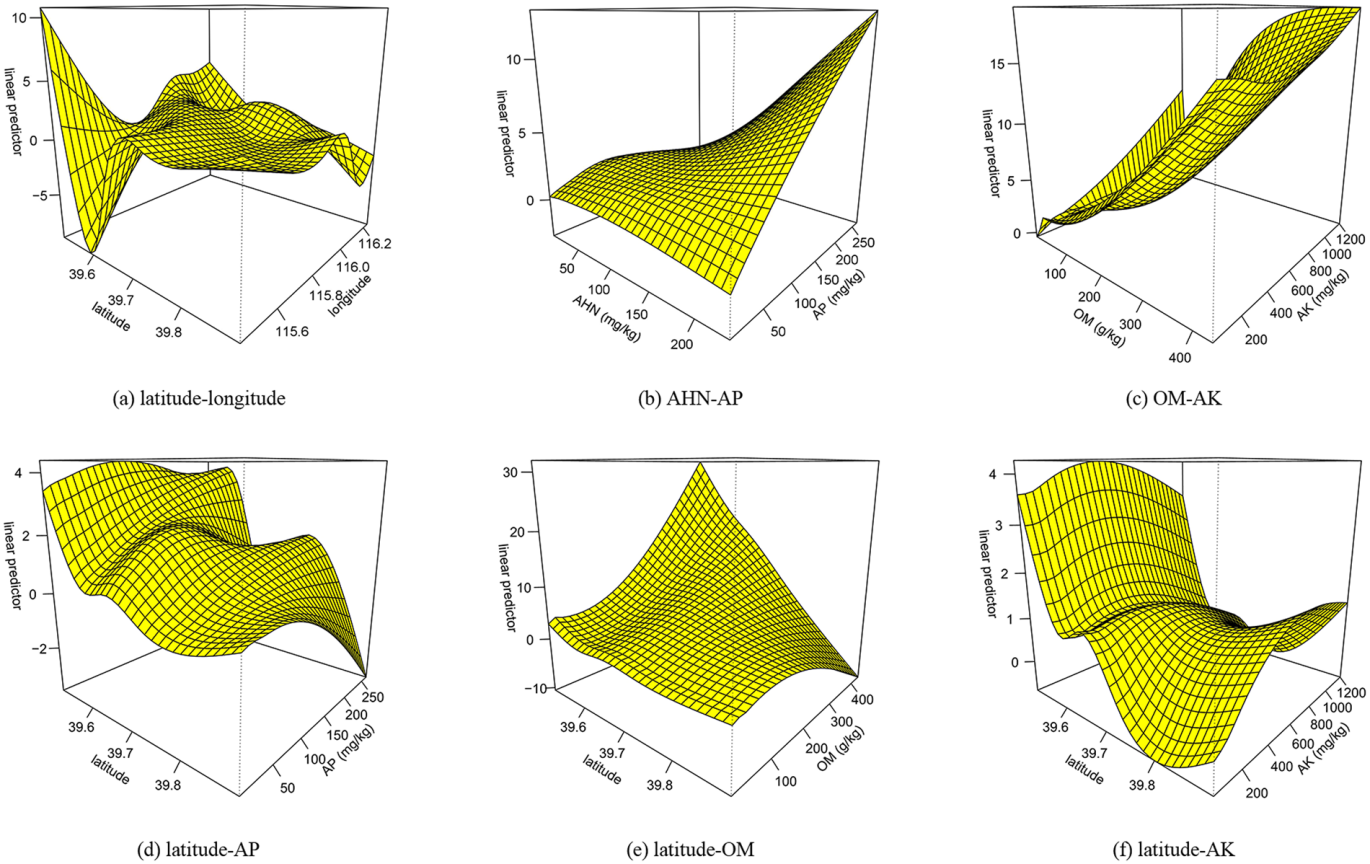
Three-dimensional effect graph of interacting influencing factors on the variation of Zn content.

The interactions that passed the significance test (*P-*value < 0.01) demonstrates the impact of interactions on Zn (Table 7 and Fig. 2). Figure 2(a) shows the influence of interaction between latitude and longitude on Zn. When latitude is less than 39.6°N, Zn decreases rapidly with the increase of longitude until it reaches at about 115.8°E. Zn reaches its local maximum at 115.8°E, 39.7°N, and then there is little increase with the increase of latitude and longitude. The influence of interaction between AHN and AP on Zn can be observed in Fig. 2(b). When AP content is less than 50 mg/kg, Zn varies little with the increase of AHN content. Above 50 mg/kg, AHN increases until AP reaches approximately 200 mg/kg until AP content reaches about 200 mg/kg. When AHN content is less than 50 mg/kg, Zn decreases with the increase of AP content. The influence of the interaction between OM and AK on Zn can be observed in Fig. 2(c). When OM content is less than 100 g/kg, Zn increase with a rise in AK. When both OM content and AK content increase, Zn increases. Figure 2(d) reveals the influence of the interaction between latitude and AP on Zn. Zn does not change significantly with the increase of AP content when the latitude is greater than 39.7°N. When the latitude is less than 39.6°N, Zn increases rapidly with the increase of AP content. Figure 2(e) shows the influence of the interaction between latitude and OM on Zn. When OM content approaches 400 g/kg, Zn decreases rapidly as latitude increases. Figure 2(f) shows the influence of the interaction between latitude and AK on Zn. The Zn content decrease with an increase in latitude when AK remains unchanged. Zinc reaches a minimum at latitude 39.8°N when AK is less than 200 mg/kg.

**Table 7:** Hypothesis test of the interaction GAM model.

Smoothing effect	EDF	Ref.df	*F-*value	*P-*value
ti(latitude)	3.929	3.993	21.049	<2e–16***
ti(longitude)	3.009	3.452	8.718	3.86e–05***
ti(OM)	2.065	2.474	2.337	0.08300
ti(AHN)	2.110	2.534	15.574	1.38e–08***
ti(AP)	2.432	2.765	43.341	<2e–16***
ti(AK)	3.008	3.337	10.724	1.76e–07***
ti(longitude, latitude)	15.281	15.762	19.857	<2e–16***
ti(longitude, AHN)	1.369	1.615	0.421	0.65992
ti(longitude, AP)	3.762	3.947	1.159	0.36403
ti(latitude, AHN)	1.000	1.000	0.079	0.77829
ti(latitude, AP)	6.157	7.034	2.534	0.01354**
ti (AP, AHN)	3.475	4.625	4.678	0.00046***
ti(latitude, OM)	4.164	5.189	2.765	0.01565**
ti(latitude, AK)	3.331	4.446	3.200	0.01036**
ti(OM, AK)	9.811	11.028	4.433	1.53e–06***
ti(longitude, OM)	1.258	1.449	1.055	0.44977
ti(longitude, AK)	1.412	1.718	0.123	0.87059
ti(OM, AHN)	5.756	6.916	1.868	0.07717
ti(AK, AHN)	2.487	3.216	1.058	0.34313

***Indicates *P-*value < 0.01 level; **indicates *P-*value < 0.05 level.

## Discussion

### Influencing factors on Zn

Latitude and longitude have significant influence on soil Zn content (*P*-value < 0.01). Conversely, Richardson *et al*.^40^ have shown that there is no correlation between site location and Zn content. The difference in results from ref.^40^ may be due to the specific geographic location and land use change in the study area. The Fangshan district is a mountainous region with manufacturing and agriculture as the prime land uses. Zinc content in soil in urban and industrial areas may be an order of magnitude greater than that in rural areas^41^. For example, Zn reaches its maximum at 115.8°E, 39.7°N (Fig. 2).

The increase of Zn due to an increase in OM and content is consistent with OM and heavy metals coexisitng in soil sediment, with OM been found to have important implications on heavy metal speciation, transport and bioavailability^42,43^. In addition, Zn content is also affected by other nutrient elements. For example, increase in flax yields in response to Zn application are most likely to occur where P fertilizer is broadcast at relatively high levels or on soils with a history of heavy P application^44^. Similarly, Zn increased as AP content increased in this study (Fig. 1).

### Modeling the Zn content

To explore the variation of Zn content in soil, the linearity of the influencing factors on Zinc were examined. On analysis of the EDFs of the smoothing functions from the univariate? GAM, it was identified that Zn content is affected by complex nonlinear influences. The univariate GAMs of Zn content in soil are able to estimate values for the significant influencing factors of latitude, longitude, OM, AHN, SAK, AP, AK. These factors are considered additive and hence a multivariate GAM was fitted, improving the goodness of fit over the univariate model. Nevertheless, SAK does not pass the significant test (*P-*value > 0.01) for the multivariate GAM but it does pass for the univariate GAM. This suggests there is a concave relationship between S(SAK) and S(AK).

Moreover, there is spatial correlation between AK and SAK in the study area. SAK refers to the potassium that exists between layers of layered silicate minerals and grain edges and cannot be reached by neutral salts in a short time. Conversely, AK can be quickly absorbed and utilized by plants. Zhang *et al*.^45^ have revealed that AK is affected more than other potassium forms and can be more sensitive in directly reflecting the productivity than SAK. On removal of SAK the goodness of fit of the multivariate GAM improved and identified that latitude, longitude, OM, AHN, AP and AK have significant influences on the Zn content in soil. Zinc content in soils is primarily affected by the interactions between latitude and OM, AP, AK (Fig. 2). The modelling suggests Zn content in soil is affected more so by the vertical direction (latitude) than the horizontal direction (longitude) in the study region. This could be due to location of manufacturing industries or natural landforms and soil types. In our study, the GAM derived from the pairwise interaction with the influencing factors can be used to analyze the influence characteristics of Zn content. Zn content is affected by multiple factors, and the interactive GAM can be constructed using three or more of these factors to analyse influences on Zn content in soil.

## Materials and Methods

### Description of the study area

Fangshan District is located between longitudes 115.4°–116.3°E and latitudes 39.5°–39.9°N in Beijing, China. It is situated to the east of the Taihang Mountains. The south-eastern region of the district is on a plain, with hill country intersecting the district from the northeast. It is in a warm temperate semi-humid monsoonal climatic zone.

### Collection of soil samples

**Figure 3:**
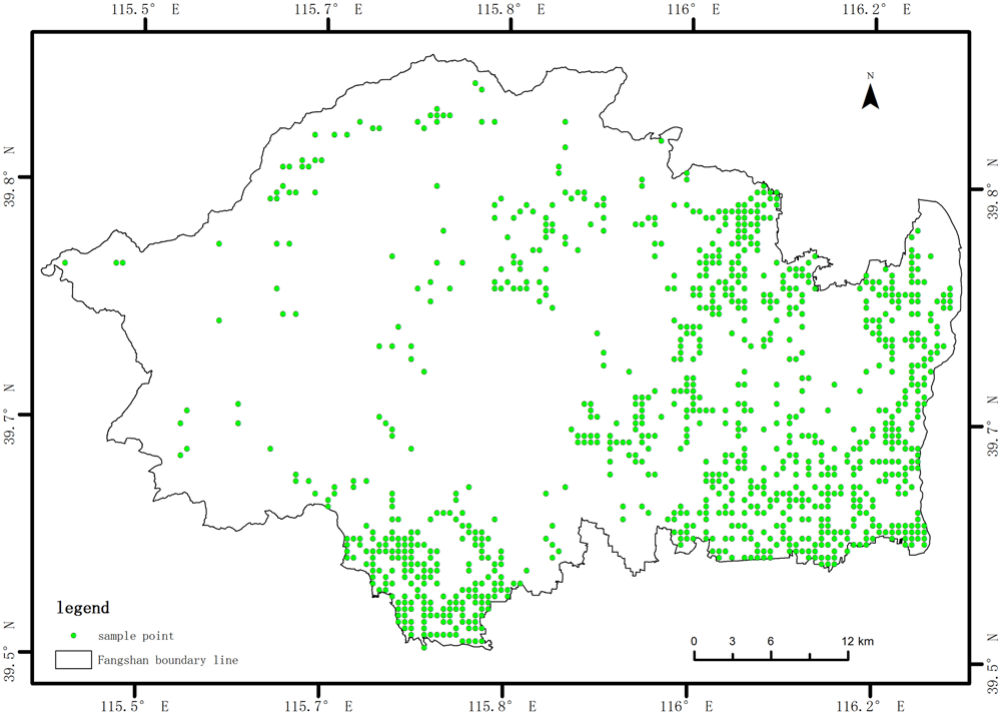
Spatial distribution of the collected soil samples in the study area.

The soil samples were primarily collected in five typical agricultural croplands including vegetable land, irrigated land, irrigated paddy field, dry land and orchards. A total of 1,497 soil samples is collected in the study area (Fig. 3). Representative soils samples were collected from random points in the croplands to a depth of 20 cm. The hybrid samples were acquired by five points and then the samples were crushed and fully mixed. Two diagonal lines were used to divide the samples into four parts. Any two parts of the diagonal angles were reserved as the final samples. A portable sub-meter GPS receiver was used to accurately acquire latitude and longitude of the sample points. Atomic Absorption Spectrometry (TAS-990, Xian Yima Optolec Co Ltd) was used to analyze the soil samples for nutrients and heavy metals. Specifically, samples were analyzed for organic matter (OM) (g/kg), alkali-hydrolyzed nitrogen (AHN) (mg/kg), available phosphorus (AP) (mg/kg), slowly available potassium (SAK) (mg/kg) and available potassium (AK) (mg/kg). Heavy metals analyzed were Zn, Fe, Cu, Mn, B and S.

### Generalized additive model

It is a regression model that can define the relationships between the response variable and each explanatory variable through smooth functions^18,31^. GAM, using an identity link function with Gaussian error distribution, is used to determine the effects of various factors on soil Zn. The generalized additive model considering interactions of two factors can be given in a general form:

1 $$g(\mu )=\sum \,{f}_{i}({X}_{i})+\sum \,{f}_{j,k}({X}_{j},{X}_{k})+\varepsilon $$

where $\mu =E(Y/{X}_{1},{X}_{2},\cdots ,{X}_{p})$; *g*(*μ*) is a link function, in this study, the log() is used as a link function; ${f}_{i}$ (*i* = 1, 2, …, 7) are the smooth functions of *X*_*i*_, *X*_*i*_ (*i* = 1, 2, …, 7) are the explanatory variables, and they are latitude, longitude, OM, AHN, AP, SAK, AK, respectively. ${f}_{j,k}()$ are the smooth functions for the interaction between these explanatory variables $({X}_{j},{X}_{k})$, $({X}_{j},{X}_{k})$ are (latitude, longitude), (AHN, AP), (OM, AK), (latitude, AP), (latitude, OM), (latitude, AK) respectively. $\varepsilon$ is the residuals and ($E(\varepsilon )=0,Var(\varepsilon )={\sigma }^{2}$).

The smooth functions with cubic regression splines were used in our work. Cubic regression splines were constructed with piecewise cubic polynomials joined together at points called knots. The definition of cubic smoothing spline basis arises from the solution of the following optimization problem. Among all the functions *f*, with two continuous derivatives, find one that minimize the penalized residual sum of squares.

2 $$\sum _{i=1}^{n}\,{\{{y}_{i}-f({x}_{i})\}}^{2}+\lambda {\int }_{a}^{b}f^{\prime\prime} {(x)}^{2}dx$$

where ${y}_{i}(i=1,2,\cdots ,n)$ is a set of observed values of the response variable and ${x}_{i}(i=1,2,\cdots ,n)$ is a set of observed values of the explanatory variable. *λ* is the smoothing parameter. $\sum _{i=1}^{n}\,{\{{y}_{i}-f({x}_{i})\}}^{2}$ measures the degree of fit of the function to the data, while $\lambda \,{\int }_{a}^{b}\,f^{\prime\prime} {(x)}^{2}dx$ adds a penalty for the curvature of the function, and the smoothing parameter controls the degree of penalty given for the curvature in the function. In our study, the position of the knots will be evenly spaced along the dimension of each explanatory variable.

### Statistical analysis

All statistical analysis in this study was undertaken in a free software environment for statistical computing and graphics (R version 3.1.2)^46^. A Shapiro-Wilk test was employed to check the normality of Zn. Correlation coefficient (*R*) was used to check the correlation between variables. In general, when there is a definite collinearity relationship between the influencing factors in the model, the concurvity relationship must exist between these factors. The existence of concurvity in GAM would not only increase the variance of coefficients but also enlarge the standard deviation of coefficients. It can cause the narrowing of confidence interval. Hence, it is necessary to test whether model has concurvity. The concurvity test has three indicators: worst, observed and estimate (Table 4). Generally, the three indicators ranging from 0 to 1 can be used to judge whether there is a concurvity. A value of 0 means no concurvity. As the test value approaches 1, the more obvious concurvity is.

### Validation of the model

A forward stepwise procedure was used to choose the most appropriate model removing each explanatory variable from the model, and then evaluating the AIC score. The smaller the score, the better the model fits. The AIC score is calculated as follows:

3 $$AIC=(2k-2L)/n$$

where *k* is the number of parameters in the model; *L* is the log likelihood; and *n* is the number of observations.

The 95% confidence interval of the fitted values for Zn was obtained from bootstrapping. Additionally, the estimated degree of freedom (EDF) was used to determine whether the selected factors were nonlinearly associated with the response variables. In order to get a reliable and stable model, a cross-validation method was used to verify the model. We randomly selected 70% of the sampling data for modeling, and the remaining 30% was used as the test set.

## Conclusions

Using the GAM, we analyzed the relationship of Zn content between latitude, longitude, OM, AHN, AK, AP and SAK in Fangshan District, Beijing. Based on our analysis, we find that Zn content in soil is significantly affected by latitude, longitude, OM, AHN, AK, AP and interactions of OM, AP, longitude, AK with latitude. Thus, by fitting a GAM, the influence of interactions between factors affecting Zn content in soil can be quantitatively predicted and analyzed. In addition, to gain a greater understanding on influencing factors on Zn content in soil, other influencing factors (e.g. pH) need to be included in the GAM.

## References

[1] Barberon M (2014). Polarization of iron-regulated transporter 1 (irt1) to the plant-soil interface plays crucial role in metal homeostasis. Proceedings of the National Academy of Sciences of the United States of America.

[2] Bouain N (2014). Phosphate and zinc transport and signaling in plants: toward a better understanding of their homeostasis interaction. Journal of Experimental Botany.

[3] Nan Z (2002). Cadmium and zinc interactions and their transfer in soil-crop system under actual field conditions. Science of the Total Environment.

[4] Ohki K (1976). Effect of zinc nutrition on photosynthesis and carbonic anhydrase activity in cotton. Physiologia Plantarum.

[5] Edillo F (2009). Effects of latitude and longitude on the population structure of Culex pipiens sl, vectors of West Nile virus in North America. The American Journal of Tropical Medicine and Hygiene.

[6] Masal E (2015). Effects of longitude, latitude and social factors on chronotype in Turkish students. Personality and Individual Differences.

[7] Rathod SR, Khedkar GD (2011). Impact of elevation, latitude and longitude on fish diversity in Godavari River. Journal of Research in Biological.

[8] Lehmann A, Overton JM, Leathwick JR (2002). GRASP: generalized regression analysis and spatial prediction. Ecological Model.

[9] Glin´Skalewczuk K (2014). Variability of zinc content in soils in a postglacial river valley–a geochemical landscape approach. Journal of Elementology.

[10] Schulz-Zunkel C (2013). Spatial and seasonal distribution of trace metals in floodplain soils. A case study with the Middle Elbe River, Germany. Geoderma.

[11] Shatar TM, McBratney AB (1999). Empirical modeling of relationships between sorghum yield and soil properties. Precision Agriculture.

[12] Kalavrouziotis IK, Koukoulakis PH (2009). Environmental implications of soil properties and essential nutrient interactions, under the effect of treated municipal wastewater. Water Air & Soil Pollution.

[13] Mendoza RE (2015). The interaction of heavy metals and nutrients present in soil and native plants with arbuscular mycorrhizae on the riverside in the Matanza-Riachuelo River Basin (Argentina). Science of the Total Environment.

[14] Liu H (2014). Grain iron and zinc concentrations of wheat and their relationships to yield in major wheat production areas in China. Field Crops Research.

[15] Hou D (2013). Distribution characteristics and potential ecological risk assessment of heavy metals (Cu, Pb, Zn, Cd) in water and sediments from Lake Dalinouer, China. Ecotoxicology and Environmental Safety.

[16] Obiakor MO, Ezeonyejiaku CD (2015). Copper-zinc coergisms and metal toxicity at predefined ratio concentrations: Predictions based on synergistic ratio mode. Ecotoxicology and Environmental Safety.

[17] Liu F (2013). Evaluation and source analysis of the mercury pollution in soils and vegetables around a large-scale zinc smelting plan. Environmental Science.

[18] Wu XL (2011). Ecological risk assessment and source analysis of heavy metals in river water, groundwater along river banks and river sediments in Shenyang. Chinese Journal of Ecology.

[19] Wenchuan Q, Dickman M, Sumin W (2001). Multivariate analysis of heavy metal and nutrient concentrations in sediments of Taihu Lake, China. Hydrobiologia.

[20] Chandrasekaran A (2015). Multivariate statistical analysis of heavy metal concentration in soils of Yelagiri Hills, Tamilnadu, India–Spectroscopical approach. Spectrochimica Acta Part A Molecular & Biomolecular Spectroscopy.

[21] Shtangeeva I (2009). Multivariate statistical analysis of nutrients and trace elements in plants and soil from northwestern Russia. Plant & Soil.

[22] Tu C, Zheng CR, Chen HM (1997). Advances on interaction of heavy metals and nutrient elements in soil-plant system. China Environmental Science.

[23] Amorós R (2018). Selenium status during pregnancy: Influential factors and effects on neuropsychological development among Spanish infants. Science of the Total Environment.

[24] Li C (2017). Modeling and projection of dengue fever cases in Guangzhou based on variation of weather factors. Science of the Total Environment.

[25] Ohshimo S (2016). Distribution, body length, and abundance of blue shark and shortfin mako offshore of northeastern Japan, as determined from observed pelagic longline data, 2000–2014. Fisheries Oceanography.

[26] Berg D (2007). Bankruptcy prediction by generalized additive models. Applied Stochastic Models in Business and Industry.

[27] Tang H (2017). The effect of environmental variables, gear design and operational parameters on sinking performance of tuna purse seine setting on free-swimming schools. Fisheries Research.

[28] Brogniez DD (2015). A map of the topsoil organic carbon content of Europe generated by a generalized additive model. European Journal of Soil Science.

[29] Ouarda TB, Charron C, Marpu PR, Chebana F (2016). The generalized additive model for the assessment of the direct, diffuse, and global solar irradiances using SEVIRI images, with application to the UAE. IEEE Journal of Selected Topics in Applied Earth Observations and Remote Sensing.

[30] Souza JBD (2018). Generalized additive models with principal component analysis: an application to time series of respiratory disease and air pollution data. Journal of the Royal Statistical Society.

[31] Yee TW, Mitchell ND (1991). Generalized additive models in plant ecology. Journal of Vegetation Science.

[32] Elith J, Leathwick JR, Hastie T (2008). A working guide to boosted regression trees. Journal of Animal Ecology.

[33] Guisan A, Edwards TC, Hastie T (2002). Generalized linear and generalized additive models in studies of species distributions: setting the scene. Ecological Model.

[34] Wood SN, Goude Y, Shaw S (2015). Generalized additive models for large data sets. Journal of the Royal Statistical Society Series C-Applied Statistics.

[35] Bishop TFA, McBratney AB (2001). A comparison of prediction methods for the creation of field-extent soil property maps. Geoderma.

[36] Jia Y (2016). Combining population growth model and generalized additive model to determine optimal water level FOR waterbird conservation: a case study of Siberian crane (*Leucogeranus Leucogeranus*) in Lake Poyang, China. River Research and Applications.

[37] Koyak RA, Hastie TJ, Tibshirani RJ (1991). Generalized Additive Models. Technometrics.

[38] Rudy ACA, Lamoureux SF, Treitz P (2016). Transferability of regional permafrost disturbance susceptibility modelling using generalized linear and generalized additive models. Geomorphology.

[39] Stone CJ (1985). Additive regression and other nonparametric models. Annals of Statistics.

[40] Richardson JB (2015). Forest floor lead, copper and zinc concentrations across the northeastern United States: synthesizing spatial and temporal responses. Science of the Total Environment.

[41] Harrison RM, Laxen DPH, Wilson SJ (1981). Chemical associations of lead, cadmium, copper, and zinc in street dusts and roadside soils. Environmental Science & Technology.

[42] Dong W (2017). Transport and humification of dissolved organic matter within a semi-arid floodplain. Journal of Environmental Sciences.

[43] Blankson ER, Adhikary NRD, Klerks PL (2017). The effect of lead contamination on bioturbation by Lumbriculus variegatus in a freshwater microcosm. Chemosphere.

[44] Grant CA, Bailey LD (1993). Interactions of zinc with banded and broadcast phosphorus fertilizer on the concentration and uptake of P, Zn, Ca and Mg in plant tissue of oil seed flax. Canadian Journal of Plant Science.

[45] Zhang JB (2012). Effects of long‐term repeated mineral and organic fertilizer applications on soil nitrogen transformations. European Journal of Soil Science.

[46] R Core Team R: A Language and Environment for Statistical Computing. *R Foundation for Statistical Computing, Vienna, Austria*. https://www.R-project.org (2014).

